# Twenty Years of Stereotype Threat Research: A Review of Psychological Mediators

**DOI:** 10.1371/journal.pone.0146487

**Published:** 2016-01-11

**Authors:** Charlotte R. Pennington, Derek Heim, Andrew R. Levy, Derek T. Larkin

**Affiliations:** Department of Psychology, Edge Hill University, Ormskirk, Lancashire, England, United Kingdom; University of Tuebingen Medical School, GERMANY

## Abstract

This systematic literature review appraises critically the mediating variables of stereotype threat. A bibliographic search was conducted across electronic databases between 1995 and 2015. The search identified 45 experiments from 38 articles and 17 unique proposed mediators that were categorized into affective/subjective (*n* = 6), cognitive (*n* = 7) and motivational mechanisms (*n* = 4). Empirical support was accrued for mediators such as anxiety, negative thinking, and mind-wandering, which are suggested to co-opt working memory resources under stereotype threat. Other research points to the assertion that stereotype threatened individuals may be motivated to disconfirm negative stereotypes, which can have a paradoxical effect of hampering performance. However, stereotype threat appears to affect diverse social groups in different ways, with no one mediator providing unequivocal empirical support. Underpinned by the multi-threat framework, the discussion postulates that different forms of stereotype threat may be mediated by distinct mechanisms.

## Introduction

The present review examines the mediators of stereotype threat that have been proposed over the past two decades. It appraises critically the underlying mechanisms of stereotype threat as a function of the type of threat primed, the population studied, and the measures utilized to examine mediation and performance outcomes. Here, we propose that one reason that has precluded studies from finding firm evidence of mediation is the appreciation of distinct forms of stereotype threat.

### Stereotype Threat: An Overview

Over the past two decades, stereotype threat has become one of the most widely researched topics in social psychology [1,2]. Reaching its 20^th^ anniversary, Steele and Aronson’s [3] original article has gathered approximately 5,000 citations and has been referred to as a 'modern classic' [4,5,6]. In stark contrast to theories of genetic intelligence [7,8] (and see [9] for debate), the theory of stereotype threat posits that stigmatized group members may underperform on diagnostic tests of ability through concerns about confirming a negative societal stereotype as self-characteristic [3]. Steele and Aronson [3] demonstrated that African American participants underperformed on a verbal reasoning test when it was presented as a diagnostic indicator of intellectual ability. Conversely, when the same test was presented as non-diagnostic of ability, they performed equivalently to their Caucasian peers. This seminal research indicates that the mere salience of negative societal stereotypes, which may magnify over time, can impede performance. The theory of stereotype threat therefore offers a situational explanation for the ongoing and intractable debate regarding the source of group differences in academic aptitude [1].

Stereotype threat has been used primarily to explain gaps in intellectual and quantitative test scores between African and European Americans [3,10] and women and men respectively [11]. However, it is important to acknowledge that many factors shape academic performance, and stereotype threat is unlikely to be the sole explanation for academic achievement gaps [12]. This is supported by research which has shown “pure” stereotype threat effects on a task in which a gender-achievement gap has not been previously documented [13], thus suggesting that performance decrements can be elicited simply by reference to a negative stereotype. Furthermore, stereotype threat effects may not be limited to social groups who routinely face stigmatizing attitudes. Rather, it can befall anyone who is a member of a group to which a negative stereotype applies [3]. For example, research indicates that Caucasian men, a group that have a relatively positive social status, underperform when they believe that their mathematical performance will be compared to that of Asian men [14]. White men also appear to perform worse than black men when motor tasks are related to 'natural athletic ability' [15,16]. From a theoretical standpoint, stereotype threat exposes how group stereotypes may shape the behavior of individuals in a way that endangers their performance and further reinforces the stereotype [10].

Over 300 experiments have illustrated the deleterious and extensive effects that stereotype threat can inflict on many different populations [17]. The possibility of confirming a negative stereotype about one’s group is found to contribute to underperformance on a range of diverse tasks including intelligence [3,13], memory [18,19], mental rotation [20–23], and math tests [11,24,25], golf putting [26], driving [27,28] and childcare skills [29]. Given the generality of these findings, researchers have turned their efforts to elucidating the underlying mechanisms of this situational phenomenon.

### Susceptibility to Stereotype Threat

Research has identified numerous moderators that make tasks more likely to elicit stereotype threat, and individuals more prone to experience it [30,31]. From a methodological perspective, stereotype threat effects tend to emerge on tasks of high difficulty and demand [32,33], however, the extent to which a task is perceived as demanding may be moderated by individual differences in working memory [34]. Additionally, stereotype threat may be more likely to occur when individuals are conscious of the stigma ascribed to their social group [32,35], believe the stereotypes about their group to be true [36,37], for those with low self-esteem [38], and an internal locus of control [39]. Research also indicates that individuals are more susceptible to stereotype threat when they identify strongly with their social group [40,41,42] and value the domain [10,13,15,33,43]. However, other research suggests that domain identification is not a prerequisite of stereotype threat effects [44] and may act as a strategy to overcome harmful academic consequences [45,46].

### Mediators of Stereotype Threat

There has also been an exPLOSion of research into the psychological mediators of stereotype threat (c.f. [2,47] for reviews). In their comprehensive review, Schmader et al. [2] proposed an integrated process model, suggesting that stereotype threat heightens physiological stress responses and influences monitoring and suppression processes to deplete working memory efficiency. This provides an important contribution to the literature, signaling that multiple affective, cognitive and motivational processes may underpin the effects of stereotype threat on performance. However, the extent to which each of these variables has garnered empirical support remains unclear. Furthermore, prior research has overlooked the existence of distinct stereotype threats in the elucidation of mediating variables. Through the lens of the multi-threat framework [31], the current review distinguishes between different stereotype threat primes, which target either the self or the social group to assess the evidence base with regards to the existence of multiple stereotype threats that may be accounted for by distinct mechanisms.

### A Multi-threat Approach to Mediation

Stereotype threat is typically viewed as a form of social identity threat: A situational predicament occurring when individuals perceive their social group to be devalued by others [48,49,50]. However, this notion overlooks how individuals may self-stigmatize and evaluate themselves [51,52,53] and the conflict people may experience between their personal and social identities [54]. More recently, researchers have distinguished between the role of the self and the social group in performance-evaluative situations [31]. The multi-threat framework [31] identifies six qualitatively distinct stereotype threats that manifest through the intersection of two dimensions: The target of the threat (i.e., is the stereotype applicable to one’s personal or social identity?) and the source of threat (i.e., who will judge performance; the in-group or the out-group?). Focusing on the target of the stereotype, individuals who experience a group-as-target threat may perceive that underperformance will confirm a negative societal stereotype regarding the abilities of their social group. Conversely, individuals who experience a self-as-target threat may perceive that stereotype-consistent performance will be viewed as self-characteristic [31,55]. Individuals may therefore experience either a self or group-based threat dependent on situational cues in the environment that heighten the contingency of a stereotyped identity [2].

Researchers also theorize that members of diverse stigmatized groups may experience different forms of stereotype threat [31,56], and that these distinct experiences may be mediated by somewhat different processes [31,57]. Indeed, there is some indirect empirical evidence to suggest that this may be the case. For example, Pavlova and colleagues [13] found that an implicit stereotype threat prime hampered women’s performance on a social cognition task. Conversely, men’s performance suffered when they were primed with an explicit gender-related stereotype. Moreover, Stone and McWhinnie [58] suggest that subtle stereotype threat cues (i.e., the gender of the experimenter) may evoke a tendency to actively monitor performance and avoid mistakes, whereas blatant stereotype threat cues (i.e., stereotype prime) create distractions that deplete working memory resources. Whilst different stereotype threat cues may simultaneously exert negative effects on performance, it is plausible that they are induced by independent mechanisms [58]. Nonetheless, insufficient evidence has prevented the multi-threat framework [31] to be evaluated empirically to date. It therefore remains to be assessed whether the same mechanisms are responsible for the effects of distinct stereotype threats on different populations and performance measures.

### Objectives

The current article offers the first systematic literature review aiming to: 1), identify and examine critically the proposed mediators of stereotype threat; 2), explore whether the effects of self-as-target or group-as-target stereotype threat on performance are the result of qualitatively distinct mediating mechanisms; and 3), evaluate whether different mediators govern different stereotyped populations.

## Method

### Literature Search

A bibliographic search of electronic databases, such as PsycINFO, PsycARTICLES, Web of Knowledge, PubMed, Science Direct and Google Scholar was conducted between the cut-off dates of 1995 (the publication year of Steele & Aronson’s seminal article) and December 2015. A search string was developed by specifying the main terms of the phenomenon under investigation. Here, the combined key words of *stereotype* and *threat* were utilized as overarching search parameters and directly paired with either one of the following terms; *mediator*, *mediating*, *mediate(s)*, *predictor*, *predicts*, *relationship* or *mechanism(s)*. Additional references were retrieved by reviewing the reference lists of relevant journal articles. To control for potential publication bias [59,60,61], the lead author also enquired about any ‘in press’ articles by sending out a call for papers through the European Association for Social Psychology. The second author conducted a comparable search using the same criteria to ensure that no studies were overlooked in the original search. Identification of relevant articles and data extraction were conducted in line with the Preferred Reporting Items for Systematic Reviews and Meta-Analyses Statement (PRISMA; See S1 Table) [62]. A literature search was conducted separately in each database and the records were exported to citation software, after which duplicates were removed. Relevant articles were screened by examining the title and abstract in line with the eligibility criteria. The remaining articles were assessed for eligibility by performing a full text review [63,64].

#### Eligibility Criteria

Studies were selected based on the following criteria: 1), researchers utilized a stereotype threat manipulation; 2), a direct mediation analysis was conducted between stereotype threat and performance; 3), researchers found evidence of moderated-mediation, and 4), the full text was available in English. Articles were excluded on the following basis: 1), performance was not the dependent variable, 2), investigations of “stereotype lift”; 3), doctorate, dissertation and review articles (to avoid duplication of included articles); and 4), moderating variables. Articles that did not find any significant results in relation to stereotype threat effects were also excluded in order to capture reliable evidence of mediation [65]. See Table 1 for details of excluded articles.

**Table 1:** Number of articles excluded in full text review, with reasons.

Reason for exclusion	Number of articles	Percentage (%)
No direct mediation analysis	25	58.14%
No ST effects found	5	11.63%
Review paper	4	9.30%
Did not prime ST	3	6.98%
Moderators of ST	3	6.98%
No performance measure	2	4.65%
Performance not standardized	1	2.33%

#### Distinguishing Different Stereotype Threats

The current review distinguished between different experiences of stereotype threat by examining each stereotype threat manipulation. Self-as-target threats were categorized on the basis that participants focused on the test as a measure of personal ability whereas group-as-target threats were classified on the basis that participants perceived performance to be diagnostic of their group’s ability [31].

### Findings

**Fig 1:**
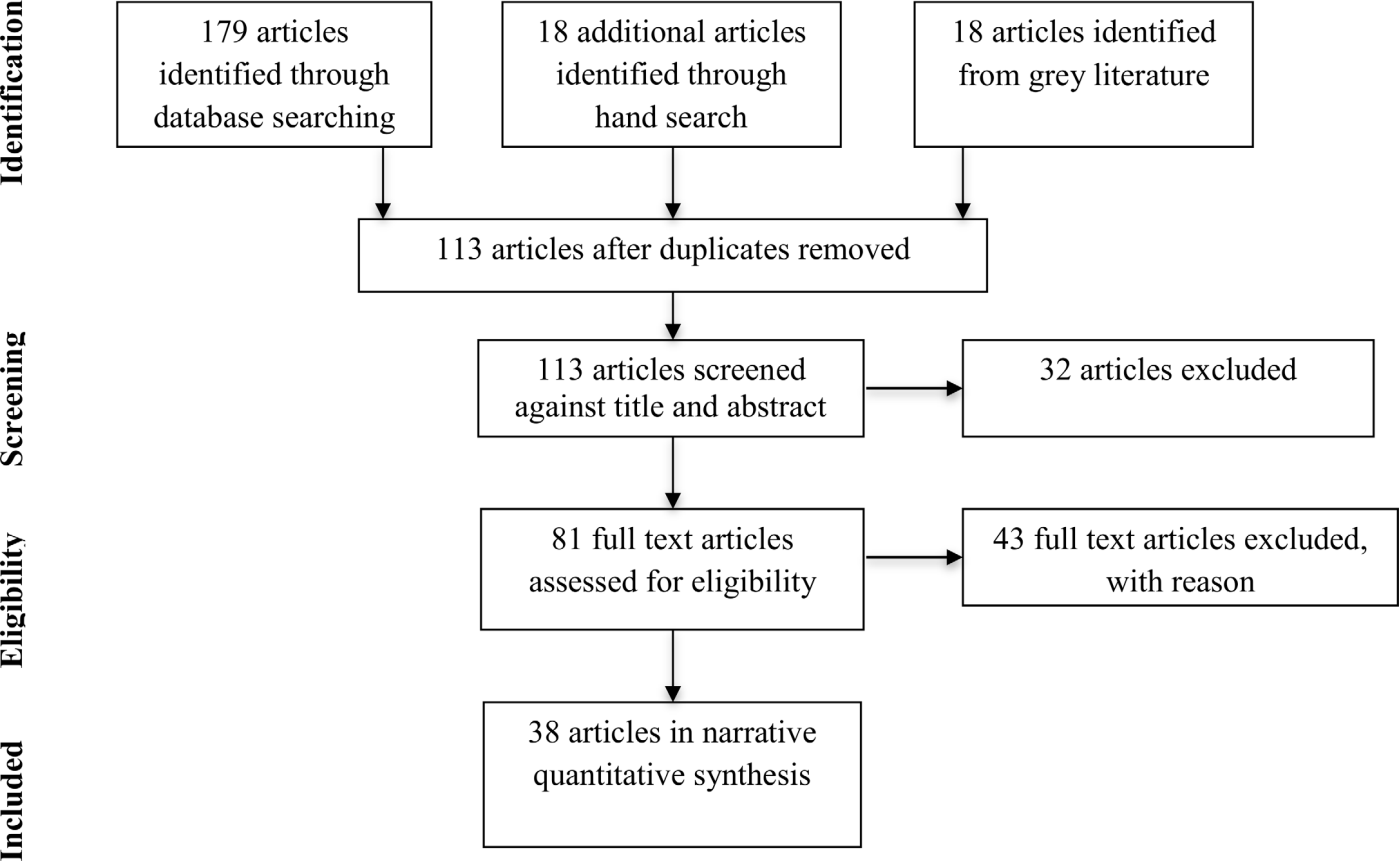
Process of article inclusion (following PRISMA).

A total of 45 studies in 38 articles were qualitatively synthesized, uncovering a total of 17 distinct proposed mediators. See Fig 1 for process of article inclusion (full details of article exclusion can be viewed in S1 Supporting Information). These mediators were categorized into affective/subjective (*n =* 6), cognitive (*n* = 7) or motivational mechanisms (*n* = 4). Effect sizes for mediational findings are described typically through informal descriptors, such as *complete*, *perfect*, or *partial* [66]. With this in mind, the current findings are reported in terms of complete or partial mediation. Complete mediation indicates that the relationship between stereotype threat (*X*) and performance (*Y*) completely disappears when a mediator (*M*) is added as a predictor variable [66]. Partial mediation refers to instances in which a significant direct effect remains between stereotype threat and performance when controlling for the mediator, suggesting that additional variables may further explain this relationship [67]. Instances of moderated mediation are also reported, which occurs when the strength of mediation is contingent on the level of a moderating variable [68]. The majority of included research utilized a group-as-target prime (*n* = 36, 80%) compared to a self-as-target prime (*n* = 6; 13.33%). Three studies (6.66%) were uncategorized as they employed subtle stereotype threat primes, for example, manipulating the group composition of the testing environment.

### Affective/Subjective Mechanisms

Researchers have conceptualized stereotype threat frequently as a fear, apprehension or anxiety of confirming a negative stereotype about one’s group [3,69,70]. Accordingly, many affective and subjective variables such as anxiety, individuation tendencies, evaluation apprehension, performance expectations, explicit stereotype endorsement and self-efficacy have been proposed to account for the stereotype threat-performance relationship.

#### Anxiety

Steele and Aronson’s [3] original study did not find self-reported anxiety to be a significant mediator of the effects of a self-relevant stereotype on African American’s intellectual performance. Extending this work, Spencer et al. (Experiment 3, [11]) found that anxiety was not predictive of the effects that a negative group stereotype had on women’s mathematical achievement, with further research confirming this [14,44,71]. Additional studies have indicated that self-reported anxiety does not influence the impact of self-as-target stereotype elicitation on African American’s cognitive ability [72], white students’ athletic skills [15], and group-as-target stereotype threat on older adults’ memory recall [18,32].

Research also suggests that anxiety may account for one of multiple mediators in the stereotype threat-performance relationship. In a field study, Chung and colleagues [73] found that self-reported state anxiety and specific self-efficacy sequentially mediated the influence of stereotype threat on African American’s promotional exam performance. This finding is supported by Mrazek et al. [74] who found that anxiety and mind-wandering sequentially mediated the effects of stereotype threat on women’s mathematical ability. Laurin [75] also found that self-reported somatic anxiety partially mediated the effects of group-as-target stereotype threat on women’s motor performance. Nevertheless, it is viable to question whether this finding is comparable to other studies as stereotype threat had a facilitating effect on performance.

The mixed results regarding anxiety as a potential mediator of performance outcomes may be indicative of various boundary conditions that enhance stereotype threat susceptibility. Consistent with this claim, Gerstenberg, Imhoff and Schmitt (Experiment 3 [76]) found that women who reported a fragile math self-concept solved fewer math problems under group-as-target stereotype threat and this susceptibility was mediated by increased anxiety. This moderated-mediation suggests that women with a low academic self-concept may be more vulnerable to stereotype threat, with anxiety underpinning its effect on mathematical performance.

Given that anxiety may be relatively difficult to detect via self-report measures [3,29], researchers have utilized indirect measures. For instance, Bosson et al. [29] found that physiological anxiety mediated the effects of stereotype threat on homosexual males’ performance on an interpersonal task. Nevertheless, this effect has not been replicated for the effects of group-as-target stereotype threat on older adults’ memory recall [32] and self-as-target threat on children’s writing ability [77].

#### Individuation tendencies

Steele and Aronson [3] proposed that stereotype threat might occur when individuals perceive a negative societal stereotype to be a true representation of personal ability. Based on this, Keller and Sekaquaptewa [78] examined whether gender-related threats (i.e., group-as-target threat) influenced women to individuate their personal identity (the self) from their social identity (female). Results revealed that participants underperformed on a spatial ability test when they perceived that they were a single in-group representative (female) in a group of males. Moreover, stereotype threat was partially mediated by ‘individuation tendencies’ in that gender-based threats influenced women to disassociate their self from the group to lessen the applicability of the stereotype. The authors suggest that this increased level of self-focused attention under solo status conditions is likely related to increased levels of anxiety.

#### Evaluation apprehension

Steele and Aronson [3] also suggested that individuals might apprehend that they will confirm a negative stereotype in the eyes of out-group members. Despite this, Mayer and Hanges [72] found that evaluation apprehension did not mediate the effects of a self-as-target stereotype threat on African American’s cognitive ability. Additional studies also indicate that evaluation apprehension does not mediate the effects of group-as-target stereotype threat on women’s mathematical performance [11,79].

#### Performance expectations

Under stereotype threat, individuals may evaluate the subjective likelihood of success depending on their personal resources. As these personal resources are typically anchored to group-level expectations, in-group threatening information (i.e., women are poor at math) may reduce personal expectancies to achieve and diminish performance [80]. Testing this prediction, Cadinu et al. (Experiment 1 [80]) found that women solved fewer math problems when they were primed with a negative group-based stereotype relative to those who received a positive or no stereotype. Furthermore, performance expectancies partially mediated the effect of group-as-target threat on math performance, revealing that negative information was associated with lower expectancies. A second experiment indicated further that performance expectancies partially mediated the effects of group-as-target threat on Black participants’ verbal ability. Research by Rosenthal, Crisp and Mein-Woei (Experiment 2 [81]) also found that performance expectancies partially mediated the effects of self-based stereotypes on women’s mathematical performance. However, rather than decreasing performance expectancies, women under stereotype threat reported higher predictions for performance relative to a control condition.

Research has extended this work to examine the role of performance expectancies in diverse stigmatized populations. For example, Hess et al. [32] found evidence of moderated-mediation for the effects of a group-as-target stereotype threat on older adults’ memory recall. Here, the degree to which performance expectancies mediated stereotype threat effects was moderated by participants’ education. That is, elderly individuals with higher levels of education showed greater susceptibility to stereotype threat. These findings add weight to the assertion that lowered performance expectations may account for the effects of stereotype threat on performance, especially among individuals who identify strongly with the ability domain. Conversely, Appel et al. [43] found that performance expectancies do not mediate the effects of group-based stereotype threat among highly identified women in the domains of science, technology, engineering and mathematics.

Further research suggests that stereotype threat can be activated through subtle cues in the environment rather than explicit stereotype activation [58,82]. It is therefore plausible that expectancies regarding performance may be further undermined when stigmatized in-group members are required to perform a stereotype-relevant task in front of out-group members. Advancing this suggestion, Sekaquaptewa and Thompson [82] examined the interactive effects of solo status and stereotype threat on women’s mathematical performance. Results revealed that women underperformed when they completed a quantitative examination in the presence of men (solo status) and under stereotype threat. However, whilst performance expectancies partially mediated the relationship between group composition and mathematical ability, they did not mediate the effects of stereotype threat on performance.

#### Explicit stereotype endorsement

Research has examined whether targeted individuals’ personal endorsement of negative stereotypes is associated with underperformance. For example, Leyens and colleagues [83] found that men underperformed on an affective task when they were told that they were not as apt as women in processing affective information. Against predictions, however, stereotype endorsement was not found to be a significant intermediary between stereotype threat and performance. Other studies also indicate that stereotype endorsement is not an underlying mechanism of the effects of self-as-target [3] and group-as-target stereotype threat on women’s mathematical aptitude [11,84].

#### Self-efficacy

Research suggests that self-efficacy can have a significant impact on an individual’s motivation and performance [85,86,87], and may be influenced by environmental cues [88]. Accordingly, it has been proposed that the situational salience of a negative stereotype may reduce an individual’s self-efficacy. As mentioned, Chung et al. [73] found that state anxiety and specific self-efficacy accounted for deficits in African American’s performance on a job promotion exam. However, additional studies indicate that self-efficacy does not mediate the effects of self-as-target threat on African American’s cognitive ability [72] and group-as-target threat on women’s mathematical performance [11].

### Cognitive Mechanisms

Much research has proposed that affective and subjective variables underpin the harmful effects that stereotype threat exerts on performance [89]. However, other research posits that stereotype threat may influence performance detriments through its demands on cognitive processes [2,89,90]. Specifically, researchers have examined whether stereotype threat is mediated by; working memory, cognitive load, thought suppression, mind-wandering, negative thinking, cognitive appraisals and implicit stereotype endorsement.

#### Working memory

Schmader and Johns [89] proposed that performance-evaluative situations might reduce working memory capacity as stereotype-related thoughts consume cognitive resources. In three studies, they examined whether working memory accounted for the influence of a group-as-target threat on women’s and Latino American’s mathematical ability. Findings indicated that both female and Latino American participants solved fewer mathematical problems compared to participants in a non-threat control condition. Furthermore, reduced working memory capacity, measured via an operation span task [91], mediated the deleterious effects of stereotype threat on math performance. Supporting this, Rydell et al. (Experiment 3 [92]) found that working memory mediated the effects of a group-relevant stereotype on women’s mathematical performance when they perceived their performance to be evaluated in line with their gender identity. Here results also showed that these performance decrements were eliminated when women were concurrently primed with a positive and negative social identity (Experiment 2).

Further research has also examined how stereotype threat may simultaneously operate through cognitive and emotional processes. Across four experiments, Johns et al. [90] found that stereotype threat was accountable for deficits in women’s verbal, intellectual and mathematical ability. Moreover, emotion regulation − characterized as response-focused coping − mediated the effects of group-as-target stereotype threat on performance by depleting executive resources.

Nonetheless, executive functioning is made up of more cognitive processes than the construct of working memory [93]. Acknowledging this, Rydell et al. [93] predicted that updating (i.e., the ability to maintain and update information in the face of interference) would mediate stereotype threat effects. They further hypothesized that inhibition (i.e., the ability to inhibit a dominant response) and shifting (i.e., people’s ability to switch between tasks) should not underpin this effect. Results indicated that women who experienced an explicit group-as-target threat displayed reduced mathematical performance compared to a control condition. Consistent with predictions, only updating mediated the stereotype threat-performance relationship. These results suggest that the verbal ruminations associated with a negative stereotype may interfere with women’s ability to maintain and update the calculations needed to solve difficult math problems.

The extent to which updating accounts for stereotype threat effects in diverse populations, however, is less straightforward. For example, Hess et al. [32] found that working memory, measured by a computational span task, did not predict the relationship between group-based stereotype threat and older participants’ memory performance.

#### Cognitive load

There is ample evidence to suggest that stereotype threat depletes performance by placing higher demands on mental resources [89,93]. These demands may exert additional peripheral activity (i.e., emotional regulation) that can further interfere with task performance [90]. In order to provide additional support for this notion, Croizet et al. [94] examined whether increased mental load, measured by participants’ heart rate, mediated the effects of stereotype threat on Psychology majors’ cognitive ability. Here, Psychology majors were primed that they had lower intelligence compared to Science majors. Results indicated that this group-as-target stereotype threat undermined Psychology majors’ cognitive ability by triggering a psychophysiological mental load. Moreover, this increased mental load mediated the effects of stereotype threat on cognitive performance.

#### Thought suppression

Research suggests that individuals who experience stereotype threat may be aware that their performance will be evaluated in terms of a negative stereotype and, resultantly, engage in efforts to disprove it [3,94,95]. This combination of awareness and avoidance may lead to attempts to suppress negative thoughts that consequently tax the cognitive resources needed to perform effectively. In four experiments, Logel et al. (Experiment 2 [95]) examined whether stereotype threat influences stereotypical thought suppression by counterbalancing whether participants completed a stereotype-relevant lexical decision task before or after a mathematical test. Results indicated that women underperformed on the test in comparison to men. Interestingly, women tended to suppress stereotypical words when the lexical decision task was administered before the math test, but showed post-suppression rebound of stereotype-relevant words when this task was completed afterwards. Mediational analyses revealed that only pre-test thought suppression partially mediated the effects of stereotype threat on performance.

#### Mind-wandering

Previous research suggests that the anticipation of a stereotype-laden test may produce a greater proportion of task-related thoughts and worries [93,95]. Less research has examined the role of thoughts unrelated to the task in hand as a potential mediator of stereotype threat effects. Directly testing this notion, Mrazek et al. (Experiment 2 [74]) found that a group-as-target stereotype threat hampered women’s mathematical performance in comparison to a control condition. Furthermore, although self-report measures of mind-wandering resulted in null findings, indirect measures revealed that women under stereotype threat showed a marked decrease in attention. Mediation analyses indicated further that stereotype threat heightened anxiety which, in turn, increased mind-wandering and contributed to the observed impairments in math performance. Despite these findings, other studies have found no indication that task irrelevant thoughts mediate the effects of group-as-target stereotype threat on women’s mathematical performance [24] and African American participants’ cognitive ability [72].

#### Negative thinking

Schmader and Johns’ [89] research suggests that the performance deficits observed under stereotype threat may be influenced by intrusive thoughts. Further research [74] has included post-experimental measures of cognitive interference to assess the activation of distracting thoughts under stereotype threat. However, the content of these measures are predetermined by the experimenter and do not allow participants to report spontaneously on their experiences under stereotype threat. Overcoming these issues, Cadinu and colleagues [96] asked women to list their current thoughts whilst taking a difficult math test under conditions of stereotype threat. Results revealed that female participants underperformed when they perceived a mathematical test to be diagnostic of gender differences. Moreover, participants in the stereotype threat condition listed more negative thoughts relative to those in the control condition, with intrusive thoughts mediating the relationship between stereotype threat and poor math performance. It seems therefore that negative performance-related thoughts may consume working memory resources to impede performance.

#### Cognitive appraisal

Other research suggests that individuals may engage in coping strategies to offset the performance implications of a negative stereotype. One indicator of coping is cognitive appraisal, whereby individuals evaluate the significance of a situation as well as their ability to control it [97]. Here, individuals may exert more effort on a task when the situational presents as a challenge, but may disengage from the task if they evaluate the situation as a threat [98,99]. Taking this into consideration, Berjot, Roland-Levy and Girault-Lidvan [100] proposed that targeted members might be more likely to perceive a negative stereotype as a threat to their group identity rather than as a challenge to disprove it. They found that North African secondary school students underperformed on a visuospatial task when they perceived French students to possess superior perceptual-motor skills. Contrary to predictions, threat appraisal did not mediate the relation between stereotype threat and performance. Rather, perceiving the situation as a challenge significantly mediated the stereotype threat-performance relationship. Specifically, participants who appraised stereotype threat as a challenge performed better than those who did not. These results therefore suggest that individuals may strive to confront, rather than avoid, intellectual challenges and modify the stereotype held by members of a relevant out-group in a favorable direction [101].

#### Implicit stereotype endorsement

Situational cues that present as a threat may increase the activation of automatic associations between a stereotyped concept (i.e., female), negative attributes (i.e., bad), and the performance domain (i.e., math; [102]). Implicit measures may be able to detect recently formed automatic associations between concepts and stereotypical attributes that are not yet available to explicitly self-report [103]. In a study of 240 six-year old children, Galdi et al. [103] examined whether implicit stereotype threat endorsement accounted for the effects of stereotype threat on girls’ mathematical performance. Consistent with the notion that automatic associations can precede conscious beliefs, results indicated that girls acquire implicit math-gender stereotypes before they emerge at an explicit level. Specifically, girls showed stereotype-consistent automatic associations between the terms ‘boy-mathematics’ and ‘girl-language’, which mediated stereotype threat effects.

### Motivational Mechanisms

Most of the initial work on the underlying mechanisms of stereotype threat has focused on affective and cognitive processes. More recently, research has begun to examine whether individuals may be motivated to disconfirm a negative stereotype, with this having a paradoxical effect of harming performance [104,105,106]. To this end, research has elucidated the potential role of effort, self-handicapping, dejection, vigilance, and achievement goals.

#### Effort/motivation

Underpinned by the “mere effort model” [104], Jamieson and Harkins [105] examined whether motivation plays a proximal role in the effect of stereotype threat on women’s math performance. Here they predicted that stereotype threat would lead participants to use a conventional problem solving approach (i.e., use known equations to compute an answer), which would facilitate performance on ‘solve’ problems, but hamper performance on ‘comparison’ problems. Results supported this hypothesis, indicating that stereotype threat debilitated performance on comparison problems as participants employed the dominant, but incorrect, solution approach. Furthermore, this incorrect solving approach mediated the effect of stereotype threat on comparison problem performance. This suggests that stereotype threat motivates participants to perform well, which increases activation of a dominant response to the task. However, as this dominant approach does not always guarantee success, the work indicates that different problem solving strategies may determine whether a person underperforms on a given task [105,107].

Stereotype threat may have differential effects on effort dependent on the prime utilized [27]. For example, Skorich et al. [27] examined whether effort mediated the effects of implicit and explicit stereotypes on provisional drivers’ performance on a hazard perception test. Participants in the implicit prime condition ticked their driving status (provisional, licensed) on a questionnaire, whereas participants in the explicit prime condition were provided with stereotypes relating to the driving ability of provisional licensees. Results revealed that participants detected more hazards when they were primed with an explicit stereotype relative to an implicit stereotype. Mediational analyses showed that whilst increased effort mediated the effects of an implicit stereotype on performance, decreased effort mediated the effects of an explicit stereotype prime. Research also indicates that reduced effort mediates the effects of an explicit stereotype on older adults’ memory recall [18]. Taken together, these results suggest that implicit stereotype primes may lead to increased effort as participants aim to disprove the stereotype, whereas explicit stereotype threat primes may lead to decreased effort as participants self-handicap [27]. Nevertheless, other studies utilizing self-reported measures of effort have resulted in non-significant findings (Experiment 1 & 2 [14]; Experiment 4 [44]; Experiment 2 [77]; Experiment 2, 4 & 5, [108]).

#### Self-handicapping

Individuals may engage in self-handicapping strategies to proactively reduce the applicability of a negative stereotype to their performance. Here, people attempt to influence attributions for performance by erecting barriers to their success. Investigating this notion, Stone [15] examined whether self-handicapping mediated the effects of stereotype threat on white athletes’ sporting performance. Self-handicapping was measured by the total amount of stereotype-relevant words completed on a word-fragment task. Results indicated that white athletes practiced less when they perceived their ability on a golf-putting task to be diagnostic of personal ability, thereby confirming a negative stereotype relating to ‘poor white athleticism’. Moreover, these athletes were more likely to complete the term ‘awkward’ on a word fragment completion test compared to the control condition. Mediation analyses revealed that the greater accessibility of the term ‘awkward’ partially mediated the effects of stereotype threat on psychological disengagement and performance. The authors suggest that stereotype threat increased the accessibility of thoughts related to poor athleticism to inhibit athletes' practice efforts. However, a limitation of this research is that analyses were based on single-item measures (i.e., the completion of the word ‘awkward’) rather than total of completed words on the word-fragment test.

Keller [109] also tested the hypothesis that the salience of a negative stereotype is related to self-handicapping tendencies. Results showed that women who were primed with a group-as-target stereotype underperformed on a mathematical test relative to their control group counterparts. Furthermore, they expressed stronger tendencies to search for external explanations for their weak performance with this mediating the effects of stereotype threat on performance. Despite these preliminary findings, Keller and Dauenheimer [44] were unable to provide support for the notion that self-reported self-handicapping is a significant intermediary between stereotype threat and women’s mathematical underperformance.

#### Dejection

Research on performance expectations suggests that stereotype threat effects may be mediated by goals set by the participants. Extending this work, Keller and Dauenheimer [44] hypothesized that female participants may make more errors on a mathematical test due to an overly motivated approach strategy. Results indicated that women underperformed when a math test was framed as diagnostic of gender differences (a group-as-target threat). Furthermore, their experiences of dejection were found to mediate the relation between stereotype threat and performance. The authors suggest that individuals may be motivated to disconfirm the negative stereotype and thus engage in a promotion focus of self-regulation. However, feelings of failure may elicit an emotional response that resultantly determines underperformance.

#### Vigilance

In contrast to Keller and Dauenheimer [44], Seibt and Förster (Experiment 5; [108]) proposed that under stereotype threat, targeted individuals engage in avoidance and vigilance strategies. They predicted that positive stereotypes should induce a promotion focus, leading to explorative and creative processing, whereas negative stereotypes should induce a prevention focus state of vigilance, with participants avoiding errors. Across five experiments, male and female participants were primed with a group-as-target stereotype suggesting that women have better verbal abilities than men. However, rather than showing a stereotype threat effect, results indicated a speed-accuracy trade off with male participants completing an analytical task slower but more accurately than their counterparts in a non-threat control condition. Furthermore, this prevention focus of vigilance was found to partially mediate the effects of stereotype threat on men’s analytical abilities (Experiment 5). The authors conclude that the salience of a negative group stereotype elicits a vigilant, risk-averse processing style that diminishes creativity and speed while bolstering analytic thinking and accuracy.

#### Achievement goals

Achievement goals theory [110] posits that participants will evaluate their role in a particular achievement context and endorse either performance-focused or performance-avoidance goals. In situations where the chances of success are low, individuals engage in performance-avoidance goals, corresponding to a desire to avoid confirming a negative stereotype. Accordingly, Chalabaev et al. [111] examined whether performance avoidance goals mediated the effects of stereotype threat on women’s sporting performance. Here, the impact of two self-as-target stereotypes (i.e., poor athletic and soccer ability) on performance were assessed relative to a control condition. Results indicated that women in the athletic ability condition performed more poorly on a dribbling task, but not in the soccer ability condition. Furthermore, although these participants endorsed a performance-avoidance goal, this did not mediate the relationship between stereotype threat and soccer performance.

Highlighting the possible interplay between affective, cognitive and motivation mechanisms, Brodish and Devine [112] proffered a multi-mediator model, proposing that anxiety and performance-avoidance goals may mediate the effects of group-as-target stereotype threat on women’s mathematical performance. Achievement goals were measured by whether participants endorsed performance-avoidant (the desire to avoid performing poorly) or approach goals (trying to outperform others). Results indicated that women under stereotype threat solved fewer mathematical problems relative to those in a control condition. Mediation analyses revealed that performance avoidance goals and anxiety sequentially mediated women’s mathematical performance. That is, stereotype threatened women were motivated to avoid failure, which in turn heightened anxiety and influenced underperformance. Table 2 summarizes the articles reviewed and details their key findings and respective methodologies. See S2 Table for overview of significant mediational findings.

**Table 2:** Summary of stereotype threat literature examining mediational variables with key methodologies and findings.

Authors	Hypothesized Mediator	Mediator Method	Dependent Variable	Population	Conditions	Stereotype threat prime	Mediation findings
Steele & Aronson [3], Experiment 2	Anxiety	State-trait anxiety index	Verbal GRE	20 black and 20 white females	2 conditions: 1), stereotype threat or control	Self-as-target	None
Spencer et al. [11], Experiment 3	Evaluation apprehension; Anxiety; Self-efficacy	State-trait anxiety index, evaluation apprehension questionnaire,	Math portion of Graduate Management Test (GMAT)	67 undergraduates (31 male)	2 conditions; 1), stereotype threat, 2), control	Group-as-target	None
Aronson et al. [14], Experiment 1	Anxiety; Effort	State-trait anxiety inventory and effort questionnaire	18 (GRE) math questions	23 male undergraduates	2 conditions: 1), stereotype threat; 2), control	Group-as-target	None
Aronson et al. [14], Experiment 2	Anxiety; Effort; Evaluation apprehension	State-trait anxiety inventory, Effort and performance expectancies questionnaire	15 GRE math questions	75 white male undergraduates	2 conditions: 1), stereotype threat; 2), control	Group-as-target	None
Stone [15]	Self-handicapping; Anxiety	Word-fragment completion task, situational anxiety questionnaire	Athletic ability; golf-putting	38 Hispanic and 36 Caucasian undergraduates	2 conditions; 1), high stereotype threat; 2), low stereotype threat	Self-as-target	Self-handicapping: Partial Anxiety: None
Hess et al. [18]	Anxiety; Effort	Memory anxiety questionnaire; strategy use (clustered recall)	30-item free recall task	48 young (22 male) and 48 older adults (25 male)	3 conditions: 1), negative stereotype; 2), positive stereotype; 3), control	Group-as-target	Anxiety: None Effort: Complete
Skorich et al. [27]	Effort	Effort measured by number of false positives on test of hazard perception	Hazard perception task	84 undergraduates (49 males)	3 conditions; 1), explicit threat; 2), categorization threat; 3), control	Self-as-target	Complete
Bosson et al. [29]	Anxiety; Evaluation apprehension	Anxiety scale Observed non-verbal anxiety	Childcare (interpersonal skills)	72 male students	2 conditions: 1), stereotype threat; 2), control	Group-as-target	Non-verbal anxiety: Complete Self-report anxiety: None
Hess et al. [32]	Working memory; Anxiety; Performance expectations	State anxiety scale and predicted recall task	Computation span task (math equations), free recall task.	103 older adults (52 male)	2 conditions; 1), stereotype threat; 2), control	Group-as-target	Performance expectations: Complete Others: None
Appel et al. [43], Experiment 4	Performance expectancies; Effort	Self-report expectancy scale; two-item self-report effort scale	Ability to judge encyclopedia entries	Female STEM majors	3 conditions: 1), stereotype threat, 2), positive stereotype, 3), control	Group-as-target	None
Keller & Dauenheimer [44]	Dejection; Anxiety; Self-handicapping	Anxiety and regulatory focus questionnaire	26 math problems	74 secondary school students (39 male)	2 conditions: 1), stereotype threat; 2), control	Group-as-target	Dejection: Complete Others: None
Tempel & Neumann, [71]	Anxiety	TAI-G anxiety questionnaire	8 arithmetic problems from the program for international student assessment	63 female undergraduates	2 conditions; 1), stereotype threat; 2), stereotype denial	Uncategorized	None
Mayer & Hanges [72]	Anxiety; Cognitive interference; Self-efficacy; Evaluation apprehension	State anxiety, self-efficacy and evaluation apprehension questionnaires,	Raven APM Cognitive test	60 African American and 90 White undergraduates (55 male)	2 conditions; 1), stereotype threat; 2), control	Self-as-target	None
Chung et al. [73]	Anxiety; Specific self-efficacy	State anxiety and self-efficacy questionnaire	Promotion performance exam	150 job applicants (134 male)	Within participants field design	Uncategorized	Complete (sequential)
Mrazek et al. [74], Experiment 2	Mind-wandering; Anxiety	Dundee State Stress questionnaire; Sustained Attention to Response Task	30 GRE math problems	72 female undergraduates	2 conditions: 1), stereotype threat; 2), control	Group-as-target	Complete (sequential)
Laurin [75]	Somatic and cognitive anxiety	Competitive state anxiety inventory and cognitive anxiety	Motor performance; 10 free throws	161 French high school students	3 conditions: 1), female stereotype threat; 2), male stereotype threat; 3), control	Group-as-target	Somatic anxiety: Partial Cognitive: None
Gerstenberg et al. [76], Experiment 3	Anxiety; Self-concept	German test anxiety scale and IAT	20 math problems	156 female undergraduates	2 conditions: 1), subtle stereotype threat; 2), control	Group-as-target	Moderated-mediation
McKown & Weinstein, [77], Experiment 2	Anxiety; Effort; Self-appraised performance	Cognitive, physiological and affective anxiety scale.	Alphabet and word task	202 elementary school children	2 conditions: 1), stereotype threat; 2), control	Self-as-target	None
Keller & Sekaquaptewa [78]	Individuation tendencies	24-item self-construal questionnaire	20-item spatial ability task	71 female students	2 conditions: 1), imagined solo status, 2), imagined non-solo status	Group-as-target	Partial
O’Brien & Crandall [79]	Evaluation apprehension	Evaluation apprehension questionnaire	3 difficult, easy and persistence math tests.	164 undergraduates (105 male)	2 conditions: 1), stereotype threat; 2), control	Group-as-target	None
Cadinu et al. [80], Experiment 1	Performance expectancies	Bar graph of performance expectancies	7 difficult math problems	95 female undergraduates	3 conditions: 1), positive stereotype, 2), negative stereotype, 3), control	Group-as-target	Partial
Cadinu et al. [80], Experiment 2	Performance expectancies	Bar graph of performance expectancies	8 sentence-completion items	100 African-American soldiers (81 male)	4 conditions: 1), American/Negative; 2), American/Positive, 3) Black/Negative; 4), Black/Positive	Group-as-target	Partial
Rosenthal et al. [81], Experiment 2	Performance expectations	Two self-report items	10 GCSE math problems	48 female undergraduates	4 shared characteristic conditions: 1), physical, 2), non-academic, 3), academic, 4), control	Group-as-target	Partial
Sekaquaptewa & Thompson, [82]	Performance expectancies	Performance expectancies questionnaire	Oral math exam	157 undergraduates (77 male)	2 conditions; 1), stereotype threat; 2), control	Group-as-target	None
Leyens et al. [83]	Explicit stereotype endorsement	Stereotype acceptance questionnaire	Lexical decision task, valence judgment task and affective decision task	50 undergraduates (26 males)	2 conditions: 1), stereotype threat; 2), control	Group-as-target	None
Beaton et al. [84]	Stereotype activation	Word-fragment completion task	9 GMAT and GRE questions	66 French-Canadian female undergraduates	3 conditions: 1), solo; 2), non-solo; 3), control	Group-as-target	None
Schmader & Johns [89], Experiment 3	Working memory	Vowel-counting and operation span task	30 GRE math problems	31 female undergraduates	2 conditions; 1), stereotype threat; 2), control	Group-as-target	Complete
Johns et al. [90], Experiment 3	Emotion regulation; Working memory	State anxiety, re-appraisal and reading-span task	30 GRE math problems	61 Caucasian female undergraduates	2 conditions: 1), stereotype threat; 2), control	Uncategorized	Complete
Rydell et al. [92], Experiment 2	Identity Accessibility	Identity accessibility task	10 GRE math problems	98 female undergraduates	4 conditions: 1), gender identity, 2), college identity, 3), multiple identities, 4), control	Group-as-target	Complete
Rydell et al. [92], Experiment 3	Working memory	Verbal vowel counting task	10 GRE math problems	57 female undergraduates	4 conditions: 1), gender identity, 2), college identity, 3), multiple identities, 4), control	Group-as-target	Complete
Rydell et al. [93], Experiment 1	Updating; Shifting; Inhibition	Stroop task, letter-memory task, number-letter task	Modular math test	168 undergraduates (93 male)	2 conditions; 1), stereotype threat; 2), control	Group-as-target	Updating: Complete Others: None
Rydell et al. [93], Experiment 2	Updating, Shifting, Inhibition	Stroop task, Keep-track task, color shape task	15 GRE Word-math problems	90 female undergraduates	2 conditions; 1), stereotype threat; 2), control	Group-as-target	Updating: Complete Others: None
Rydell et al. [93], Experiment 3	Updating, Shifting, Inhibition	Letter-memory task, color-shape task, anti-saccade task.	GRE Word-math problems	82 female undergraduates	2 conditions; 1), stereotype threat; 2), control	Group-as-target	Updating: Complete Others: None
Croizet et al. [94]	Increased mental load	Heart rate variability	Raven APM cognitive test	139 college students	2 conditions: 1), stereotype threat; 2), control	Group-as-target	Complete
Logel et al. [95], Experiment 2	Thought suppression	Lexical decision task	20 math problems	71 undergraduates (35 male)	2 conditions: 1), subtle stereotype threat; 2), control	Group-as-target	Partial
Cadinu et al. [96]	Negative thinking	Thought-listing sentences	7 GRE math problems	60 female undergraduates	2 conditions; 1), stereotype threat; 2), control	Group-as-target	Complete
Berjot et al. [100]	Cognitive appraisals (challenge)	State primary appraisal questionnaire	Visuospatial performance: Ray figure	92 French secondary school students (53 male)	2 conditions: 1), stereotype threat; 2), control	Group-as-target	Complete
Galdi et al. [103]	Implicit stereotype endorsement	Implicit Association Test (IAT)	Math test	276 first grade children (133 male)	3 conditions: 1), stereotype-consistent; 2), inconsistent; 3), control	Group-as-target	Complete
Jamieson & Harkins [105]	Effort	Coded solving techniques	30 GRE math problems	76 female undergraduates	2 conditions; 1), stereotype threat; 2), control	Group-as-target	Complete
Seibt & Förster [108], Experiment 2	Motivation; Expectancy	Motivation and performance expectancies questionnaire	Word-selection task	60 undergraduates students (29 male)	2 conditions; 1), stereotype threat; 2), control	Group-as-target	None
Seibt & Förster [108], Experiment 4	Motivation, expectancy; Mood; Liking for task	Motivation, expectancies, mood and liking questionnaires	4 reasoning GRE problems and brick task	28 German undergraduates	2 conditions: 1), positive stereotype; 2), negative stereotype	Group-as-target	None
Seibt & Förster [108], Experiment 5	Vigilance; Motivation; Expectancy; Mood; Liking of the task	Self-report eagerness and vigilance strategies, motivation and expectancy questionnaire	Analytic reasoning GRE problems and categorization task	42 undergraduates	3 conditions; 1), positive stereotype; 2), negative stereotype; 3), control	Group-as-target	Vigilance: Partial
Keller [109]	Self-handicapping	2-item self-handicapping questionnaire	20 math problems	75 German secondary school students	2 conditions: 1), stereotype threat; 2), control	Group-as-target	Dejection: Complete Others: None
Chalabaev et al. [111]	Achievement goals	Achievement goals questionnaire for sports	Ability to dribble soccer ball through slalom course	51 female soccer players	3 conditions: 1), Athletic ability stereotype threat; 2), Technical ability stereotype threat; 3), control	Self-as-target	None
Brodish & Devine [112]	Performance Goals; Anxiety	State anxiety and performance goals scale	20 GRE math problems	101 female undergraduates	2 conditions: 1), stereotype threat; 2), control	Group-as-target	Complete

## Discussion

The current review evaluated empirical support for the mediators of stereotype threat. Capitalizing on the multi-threat framework [31], we distinguished between self-relevant and group-relevant stereotype threats to examine the extent to which these are mediated by qualitatively distinct mechanisms and imperil diverse stigmatized populations. On the whole, the results of the current review indicate that experiences of stereotype threat may increase individuals’ feelings of anxiety, negative thinking and mind-wandering which deplete the working memory resources required for successful task execution. Research documents further that individuals may be motivated to disconfirm the negative stereotype and engage in efforts to suppress stereotypical thoughts that are inconsistent with task goals. However, many of the mediators tested have resulted in varying degrees of empirical support. Below we suggest that stereotype threat may operate in distinct ways dependent on the population under study, the primes utilized, and the instruments used to measure mediation and performance.

Previous research has largely conceptualized stereotype threat as a singular construct, experienced similarly by individuals and groups across situations [31,55]. Consequently, research has overlooked the possibility of multiple forms of stereotype threats that may be implicated through concerns to an individual’s personal *or* social identity [31]. This is highlighted in the present review, as the majority of stereotype threat studies employed a group-as-target prime. Here stereotype threat is typically instantiated to highlight that stereotype-consistent performance may confirm, or reinforce, a negative societal stereotype as being a true representation of one’s social group [48]. This has led to a relative neglect of situations in which individuals may anticipate that their performance may be indicative of personal ability [31,55].

Similar processes such as arousal, deficits in working memory, and motivation may be triggered by self-as-target and group-as-target stereotype threats. However, it is important to note that the experiences of these stereotype threats may be fundamentally distinct [31]. That is, deficits in working memory under self-as-target stereotype threat may be evoked by negative thoughts relating to the self (i.e., ruining one’s opportunities, letting oneself down). Conversely, group-based intrusive thoughts may mediate the effects of group-as-target threat on performance as individuals view their performance in line with their social group (i.e., confirming a societal stereotype, letting the group down) [31]. Moreover, research suggests that when a group-based stereotype threat is primed, individuals dissociate their sense of self from the negatively stereotyped domain [78]. Yet, this may be more unlikely when an individual experiences self-as-target stereotype threat as their personal ability is explicitly tied to a negative stereotype that governs their ingroup. As such, the activation of a group-based stereotype may set in motion mechanisms that reflect a protective orientation of self-regulation, whereas self-relevant knowledge may heighten self-consciousness. To date, however, research has not explicitly distinguished between self-as-target and group-as-target stereotype threat in the elucidation of mediating variables. Future research would therefore benefit from a systematic investigation of how different stereotype threats may hamper performance in qualitatively distinguishable ways. One way to investigate the hypotheses set out here would be to allow participants to spontaneously report their experiences under self-as-target and group-as-target stereotype threat, and to examine differences in the content of participants’ thoughts as a function of these different primes.

In a similar vein, different mechanisms may mediate the effects of blatant and subtle stereotype threat effects on performance [27,58,111]. Blatant threat manipulations explicitly inform participants of a negative stereotype related to performance (e.g., [3,11]), whereas placing stigmatized group members in a situation in which they have minority status may evoke more subtle stereotype threat [78,82]. Providing evidence consistent with this notion, Sekaquaptewa and Thompson [82] found that performance expectancies partially mediated the effects of solo status, but not stereotype threat on performance. These results suggest that women may make comparative judgments about their expected performance when they are required to undertake an exam in the presence of out-group members, yet may not consciously recognize how a negative stereotype can directly impair performance. Further research suggests that working memory may mediate the effects of subtle stereotype threat cues on performance as individuals attend to situational cues that heighten the salience of a discredited identity [88,94]. Alternatively, motivation may mediate the effects of blatant stereotype threat as individuals strive to disprove the negative stereotype [27,44,58,108]. Although stereotype threat effects appear to be robust [30], it is plausible that these distinct manipulations diverge in the nature, the focus, and the intensity of threat they produce and may therefore be mediated by different mechanisms [31].

It is also conceivable that different groups are more susceptible to certain types of stereotype threat [13,31,56]. For example, research indicates that women’s performance on a social cognition task was influenced to a greater extent by implicit gender-related stereotypes, whereas men were more vulnerable to explicit stereotype threat [13]. Further research suggests that populations who tend to have low group identification (e.g., those with a mental illness or obesity) are more susceptible to self-as-target threats. Conversely, populations with high group identification, such as individuals of a certain ethnicity, gender or religion are more likely to experience group-as-target threats [56]. Whilst this highlights the role of moderating variables that heighten individuals’ susceptibility to stereotype threat, it also suggests that individuals may experience stereotype threat in different ways, dependent on their stigmatized identity. This may explain why some variables (e.g., anxiety, self-handicapping) that have been found to mediate the effects of stereotype threat on some groups have not emerged in other populations.

Finally, it is conceivable that diverse mediators account for the effects of stereotype threat on different performance outcomes. For example, although working memory is implicated in tasks that typically require controlled processing, it is not required for tasks that rely more on automatic processes [24,58,93]. In line with this notion, Beilock et al. [24] found that experts’ golf putting skills were harmed under stereotype threat when attention was allocated to automatic processes that operate usually outside of working memory. This suggests that well-learned skills may be hampered by attempts to bring performance back under step-by-step control. Conversely, skills such as difficult math problem solving appear to involve heavy processing demands and may be harmed when working memory is consumed by a negative stereotype. As such, distinct mechanisms may underpin different threat-related performance outcomes.

### Limitations of Stereotype Threat Research

We now outline methodological issues in current stereotype threat literature with a view to inform the design of future research. First, researchers have predominantly utilized self-report measures in their efforts to uncover the mediating variables of stereotype threat. However, it has long been argued that individuals have limited access to higher order mental processes [113,114], such as those involved in the evaluation and initiation of behavior [115,116]. Resultantly, participants under stereotype threat may be unable to observe and explicitly report the operations of their own mind [29,114,117,118,119]. Consistent with this assertion, Bosson et al. [29] found that although stereotype threat heightened individuals’ physiological anxiety, the same individuals did not report an awareness of increased anxiety on self-report measures. Participants may thus be mindful of the impression they make on others and engage in self-presentational behaviors in an effort to appear invulnerable to negative stereotypes [29]. This is supported by research suggesting that stereotype threatened participants tend not to explicitly endorse stereotypes [29,37,83,84] and are more likely to claim impediments to justify poor performance [3,14,109]. Moreover, it is possible that stereotype threat processes are non-conscious [119] with research indicating that implicit–but not explicit–stereotype endorsement mediates stereotype threat effects [103]. This suggests that non-conscious processing of stereotype-relevant information may influence the decrements observed in individuals’ performance under stereotype threat. Furthermore, this research underscores the greater sensitivity of indirect measures for examining the mediators of stereotype threat. From this perspective, future research may benefit from the use of physiological measures, such as heart rate, cortisol and skin conductance to examine anxiety (c.f., [94,120,121]), the IAT to measure implicit stereotype endorsement [103] and the sustained response to attention task to measure mind-wandering [74].

In the investigation of stereotype threat, self-report measures may be particularly susceptible to order effects. For example, Brodish and Devine [112] found that women reported higher levels of anxiety when they completed a questionnaire before a mathematical test compared to afterwards. This suggests that pre-test anxiety ratings may have reflected participants’ uneasiness towards the upcoming evaluative test, with this apprehension diminishing once the test was completed. Research by Logel and colleagues [95] provides support for this notion, indicating that women who completed a lexical decision task after a math test were quicker to respond to stereotype-relevant words compared to women who subsequently completed the task. These results exhibit the variability in individuals’ emotions under stereotype threat and suggest that they may be unable to retrospectively report on their feelings once the threat has passed. This emphasizes the importance of counterbalancing test instruments in the investigation of stereotype threat, purporting that the order in which test materials are administered may influence mediational findings.

This review highlights that, in some studies, individuals assigned to a control condition may have also experienced stereotype threat, thus potentially preventing reliable evidence of mediation. For instance, Chalabaev et al. [111] primed stereotype threat by presenting a soccer ability test as a diagnostic indicator of personal factors related to athletic ability. Nevertheless, participants in the control condition received information that the aim of the test was to examine psychological factors in athletic ability. Consequently, these participants may have also been apprehensive about their performance being evaluated, and this may have precluded evidence that achievement goals mediate the stereotype threat-performance relationship. Furthermore, research has manipulated the salience of stereotype threat by stating that gender differences in math performance are equal [82]. However, other research has utilized this prime within control conditions (e.g., [94,105,119]), underpinned by the rationale that describing a test as ‘fair’ or non-diagnostic of ability eliminates stereotype threat [122]. It is therefore possible that, in some instances, researchers have inadvertently induced stereotype threat. This outlines the importance of employing a control condition in which individuals are not made aware of any negative stereotypes, and are told that the test is non-diagnostic of ability, in order to detect possible mediators.

### Conclusion

Two decades of research have demonstrated the harmful effects that stereotype threat can exert on a wide range of populations in a broad array of performance domains. However, findings with regards to the mediators that underpin these effects are equivocal. This may be a consequence of the heterogeneity of primes used to instantiate stereotype threat and the methods used to measure mediation and performance. To this end, future work is likely to benefit from the following directions: First, account for the existence of multiple stereotype threats; Second, recognize that the experiences of stereotype threat may differ between stigmatized groups, and that no one mediator may provide generalized empirical support across diverse populations; Third, utilize indirect measures, in addition to self-report measures, to examine reliably mediating variables and to examine further the convergence of these two methods; Fourth, counterbalance test instruments to control for order effects; and finally, ensure that participants in a control condition do not inadvertently encounter stereotype threat by stating explicitly that the task is non-diagnostic of ability.

## Supporting Information

S1 Supporting Information List of excluded studies and rationale for exclusion. (DOCX)

S1 Table PRISMA Checklist. (DOC)

S2 Table Summary of affective, cognitive and motivational mechanisms that have been found to mediate stereotype threat effects. (DOCX)
