# Supplementary material for: Twenty Years of Stereotype Threat Research: A Review of Psychological Mediators
Source: PLoS One. 2016 Jan 11;11(1):e0146487. doi: 10.1371/journal.pone.0146487 (PMC4713435; doi:10.1371/journal.pone.0146487)
Supplement: S1 Supporting Information — (DOCX) [file pone.0146487.s001.docx]

**S1. Supporting information**

**List of included articles**

**The following 38 articles were included in the systematic review:**

Appel M, Kronberger N, Aronson J. Stereotype threat impairs ability building: Effects on test preparation among women in science and technology. Eur J Soc Psychol. 2011;41: 904-913. doi:10.1002/ejsp.835

Aronson J, Lustina MJ, Good C, Keough K, Steele CM, Brown J. When white men can’t do math: Necessary and sufficient factors in stereotype threat. J Exp Soc Psychol. 1999;35: 29-46. doi:10.1006/jesp.1998.1371

Beaton A, Tougas F, Rinfret N, Huard N, Delisle M-N. Strength in numbers? Women and mathematics. Eur J of Psychol Educ. 2009;22: 291-306. doi:10.1007/BF03173427

Berjot S, Roland-Levy C, Girault-Lidvan N. Cognitive appraisals of stereotype threat. Psychol Rep. 2011;108: 585-598. doi:10.2466/04.07.21.PR0.108.2.585-598

Bosson JK, Haymovitz EL, Pinel EC. When saying and doing diverge: The effect of stereotype threat on self-reported versus non-verbal anxiety. J Exp Soc Psychol. 2004;40: 247-255. doi:10.1016/S0022-1031(03)00099-4

Brodish AB, Devine, PG. The role of performance-avoidance goals and worry in mediating the relationship between stereotype threat and performance. J Exp Soc Psychol. 2009;45: 180-185. doi:10.1016/j.jesp.2008.08.005

Cadinu M, Maass A, Frigerio S, Impagliazzo L, Latinotti S. Stereotype threat: The effect of expectancy on performance. Eur J Soc Psychol. 2003;33: 267-285. doi:10.1002/ejsp.145

Cadinu M, Maass A, Rosabianca A, Kiesner J. Why do women underperform under stereotype threat? Evidence for the role of negative thinking. Psychol Sci. 2005;16: 572, 578. doi:10.1111/j.0956-7976.2005.01577.x

Chalabaev A, Sarrazin P, Stone J, Cury F. Do achievement goals mediate stereotype threat? An investigation on females’ soccer performance. J Sport Exerc Psychol. 2008;20: 143-158.

Chung BG, Ehrhart MG, Holcombe-Ehrhart K, Hattrup K, Solamon J. Stereotype threat, state anxiety, and specific self-efficacy as predictors of promotion exam performance. Group Organ Manage. 2010;35: 77-107. doi:10.1177/1059601109354839

Croizet J-C, Després G, Gauzins M-E, Huguet P, Leyens J-P, Méot, A. Stereotype threat undermines intellectual performance by triggering a disruptive mental load. Pers Soc Psychol Bull. 2004;30: 721-731. doi:10.1177/0146167204263961

Galdi S, Cadinu M, Tomasetto C. The roots of stereotype threat: When automatic associations disrupt girls’ math performance. Child Dev. 2014;85: 250-263. doi:10.1111/cdev.12128

Gerstenberg FXR, Imhoff R, Schmitt M. ‘Women are bad at math, but I’m not, am I?’ Fragile mathematical self-concept predicts vulnerability to a stereotype threat effect on mathematical performance. Eur J Pers. 2012;26: 588-599. doi:10.1002/per.1836

Hess TM, Auman C, Colcombe SJ, Rahhal TA. The impact of stereotype threat on age differences in memory performance. J Gerontol B Psychol Sci Soc Sci. 2003;58: 3-11. doi:10.1093/geronb/58.1.P3

Hess TM, Hinson, JT, Hodges, EA. Moderators of and mechanisms underlying stereotype threat effects on older adults’ memory performance. Exp Aging Res. 2009;35: 153-177. doi:10.1080/03610730802716413

Jamieson JP, Harkins SG. Distinguishing between the effects of stereotype priming and stereotype threat on math performance. Group Process Intergroup Relat. 2011;15: 291-304. doi:10.1177/1368430211417833

Johns M, Inzlicht M, Schmader T. Stereotype threat and executive resource depletion: Examining the influence of emotion regulation. J Exp Psychol: Gen. 2008;137: 691-705. doi:10.1037/a0013834

Keller J, Dauenheimer D. Stereotype threat in the classroom: Dejection mediates the disrupting effect of women’s math performance. Pers Soc Psychol Bull. 2003;29: 371-381. doi:10.1177/0146167202250218

Keller J, Sekaquaptewa D. Solo status and women’s spatial test performance: The role of individuation tendencies. Eur J Soc Psychol. 2008;38: 1044-1053. doi:10.1002/ejsp.490

Keller J. Blatant stereotype threat and women’s math performance: Self-handicapping as a strategic means to cope with obtrusive negative performance expectations. Sex Roles. 2002;47: 193-198. doi:10.1023/A:1021003307511

Laurin R. Stereotype threat and lift effects in motor task performance: The mediating role of somatic and cognitive anxiety. J Soc Psychol. 2013;153: 687-699. doi:10.1080/00224545.2013.821098

Leyens J-P, Désert M, Croizet J-C, Darcis C. Stereotype threat: Are lower status and history of stigmatization preconditions of stereotype threat? Pers Soc Psychol Bull. 2000;26: 1189-1199. doi:10.1177/0146167200262002

Logel C, Iserman EC, Davies PG, Quinn DM, Spencer SJ. The perils of double consciousness: The role of thought suppression in stereotype threat. J Exp Soc Psychol. 2009;45: 299-312. doi:10.1016/j.jesp.2008.07.016

Mayer DM, Hanges PJ. Understanding the stereotype threat effect with “culture free” tests: An examination of its mediators and measurement. Hum Perf. 2003;16: 207-230. doi:10.1207/S15327043HUP1603_3

McKown C, Weinstein RS. The development and consequences of stereotype consciousness in middle childhood. Child Dev. 2003;74: 498-515. doi:10.1111/1467-8624.7402012

Mrazek MD, Chin JM, Schmader T, Hartson KA, Smallwood J, Schooler JW. Threatened to distraction: Mind-wandering as a consequence of stereotype threat. J Exp Soc Psychol. 2011;47: 1243-1248. doi:10.1016/j.jesp.2011.05.011

O’Brien LT, Crandall CS. Stereotype threat and arousal: Effects on women’s maths performance. Pers Soc Psychol Bull. 2003;29: 782-789. doi:10.1177/0146167203029006010

Rosenthal HES, Crisp RJ, Suen M-W. Improving performance expectancies in stereotypic domains: Task relevance and the reduction of stereotype threat. Eur J Soc Psychol. 2007:37; 586-597. doi:10.1002/ejsp.379

Rydell RJ, McConnelL AR, Beilock SL. Multiple social identities and stereotype threat: Imbalance, accessibility, and working memory. J Pers Soc Psychol. 2009;96: 949-966. doi:10.1037/a0014846.

Rydell RJ, Van Loo KJ, Boucher KL. Stereotype threat and executive functions: Which functions mediate different threat-related outcomes? Pers Soc Psychol Bull. 2014;40: 377-390. doi:10.1177/0146167213513475

Schmader T, Johns M. Converging evidence that stereotype threat reduces working memory capacity. J Pers Soc Psychol. 2003;85: 440-452. doi:10.1037/0022-3514.85.3.440

Seibt B, Förster J. Stereotype threat and performance: How self-stereotypes influence processing by inducing regulatory foci. J Pers Soc Psychol. 2004;87: 38-56. doi:10.1037/0022-3514.87.1.38

Sekaquaptewa D, Thompson M. Solo status, stereotype threat, and performance expectancies: Their effects on women’s performance. J Exp Soc Psychol. 2003;39: 68-74. doi:10.1016/S0022-1031(02)00508-5

Skorich DP, Webb H, Stewart L, Kostyanaya M, Cruwyz T, McNeill K, et al. Stereotype threat and hazard perception among provisional license drivers. Accid Anal Prev. 2013;54: 39-45. doi:10.1016/j.aap.2013.02.002

Spencer SJ, Steele CM, Quinn DM. Stereotype threat and women’s math performance. J Exp Soc Psychol. 1999;35: 4–28. doi:10.1006/jesp.1998.1373

Steele CM, Aronson J. Stereotype threat and the intellectual test performance of African Americans. J Pers Soc Psychol. 1995;69: 797-811. doi:10.1037/0022-3514/0022-3514.69.5.797

Stone J. Battling doubt by avoiding practice: The effects of stereotype threat on self-handicapping in White athletes. Pers Soc Psychol Bull. 2002;28: 1667-1678. doi:10.1177/014616702237648

Tempel T, Neumann R. Stereotype threat, test anxiety, and mathematics performance. Soc Psychol Educ. 2014;17: 491-501. doi:10.1007/s11218-014-9263-9

**List of excluded articles and rationale for exclusion**

**The following 25 articles were excluded as they did not conduct a mediational analysis or did conduct a direct mediational analysis between stereotype threat and performance:**

Allison ES, Harkins SG. Stereotype threat, mental arithmetic, and the mere effort account. Journal of Experimental Social Psychology. 2015:61; 19-30.

Barber SJ, Mather M. Stereotype threat can both enhance and impair older adults’ memory. Psychological Science. 2013:24; 2522-2529.

Beilock SL, Jellison WA, Rydell RJ, McConnell AR, Carr TH. Mechanisms of stereotype threat: Can skills that don’t rely heavily on working memory still be threatened? Personality and Social Psychology Bulletin. 2006:32; 1059-1071.

Beilock SL, Rydell RJ, McConnell AR. Stereotype threat and working memory: Mechanisms, alleviation and spillover. Journal of Experimental Psychology: General. 2007:136; 256-276.

Ben-Zeev T, Fein S, Inzlicht M. Arousal and stereotype threat. Journal of Experimental Social Psychology. 2005:41; 174-181.

Blascovich J, Spencer SJ, Quinn D, Steele C. African Americans and high blood pressure: The role of stereotype threat. Psychological Science. 2001:12; 225-229.

Chasteen AL, Bhattacharyya S, Horhota M, Tam R, Hasher L. How feelings of stereotype threat influence older adults’ memory performance. Experimental Aging Research. 2005:31; 235-260.

Croizet JC, Després G, Gauzins ME, Huguet P, Leyens JP, Mêot A. Stereotype threa undermines intellectual performance by triggering a disruptive mental load. Personality and Social Psychology Bulletin. 2004:30; 721-731.

Feltz DL, Schneider R, Hwang S, Skogsberg NJ. Predictors of collegiate student-athletes’ susceptibility to stereotype threat. Journal of College Student Development. 2013:54; 184-201.

Fogliati VJ, Bussey K. Stereotype threat reduces motivation to improve: Effects of stereotype threat and feedback on women’s intentions to improve mathematical ability. Psychology of Women Quarterly. 2013;37: 310-324.

Franceschini G, Galli S, Chiesi F, Primi C. Implicit gender-math stereotype and women’s susceptible to stereotype threat and stereotype lift. Learning and Individual Differences. 2014:32; 273-277.

Huber ME, Seitchik AE, Brown AJ, Sternad D, Harkins SG. The effect of stereotype threat on performance of a rhythmic motor skill. Journal of Experimental Psychology: Human Perception and Performance. 2015:41; 525-541.

Jamieson JP, Harkins SG. Mere effort and stereotype threat performance effects. Journal of Personality and Social Psychology. 2007:93: 544-564.

Krendl AC, Richeson JA, Kelley WM, Heatherton TF. The negative consequences of stereotype threat: Functional magnetic resonance imaging investigation of the neutral mechanisms underlying women’s underperformance in math. Psychological Science. 2008:19; 168-175.

Lawrence JS, Marks BT, Jackson JS. Domain identification predicts black students’ underperformance on moderately-difficult tests. Motivation & Emotion. 2010:34: 105-109.

Leitner JB, Johns JM, Hehman E. Succeeding in the face of stereotype threat: The adaptive role of engagement regulation. Personality and Social Psychology Bulletin. 2013:39; 17-27.

Massey DS, Owens J. Mediators of stereotype threat mong black college students. Ethnic and Racial Studies. 2014:37; 556-575.

Mazerolle M, Régner I, Morisset P, Rigalleau F, Huguet P. Stereotype threat strengthens automatic recall and undermines controlled processes in older adults. Psychological Science. 2012;23: 723-727.

Osborne JW. Linking stereotype threat and anxiety. Educational Psychology. 2007:27; 135-154.

Rydell RJ, Shiffrin RM, Boucher KL, Van-Loo K, Rydell MT. Stereotype threat prevents perceptual learning. PNAS. 2010:107; 14042-14047.

Schmader T, Croft A, Whitehead J. Why can’t I just be myself?: A social cognitive analysis of the working self-concept under stereotype threat. Psychology and Personality Science. 2014:5; 4-11.

Schmader T, Johns M, Forbes C. An intergrated process model of stereotype threat effects on performance. Psychological Review. 2008:115; 336-356.

Schweinle A, Mims GA. Mathematics self-efficacy: Stereotype threat versus resilience. Social Psychology of Education. 2009:12; 501-514.

Stone J, Lynch CI, Sjomeling M, Darley JM. Stereotype threat effects on Black and White athletic performance. Journal of Personality and Social Psychology. 1999:77; 1213-1227.

Tine M, Gotlieb R. Gender-, race-, and income-based stereotype threat: The effects of multiple stigmatized aspects of identity on math performance and working memory function. Social Psychology of Education. 2013:16; 353-376.

**The following 5 articles were excluded, as they did not find stereotype threat effects:**

Ganley CM, Mingle LA, Ryan AM, Ryan K, Vasilyeva M, Perry M. An examination of stereotype threat effects on girls’ mathematics performance. Developmental Psychology. 2013:49; 1886-1897.

Kellow JT, Jones BD. The effects of stereotypes on the achievement gap: Reexamining the academic performance of African American high school students. Journal of Black Psychology. 2008:34; 94-120.

Lee J-E, R, Nass CI, Bailenson JN. Does the mask govern the mind?: Effects of arbitrary gender representation on quantitative task performance in avatar-represented virtual groups. Cyberpsychology, Behavior, and Social Networking. 2014:17; 248-254.

Nguyen H-H, O’Neil A, Ryan AM. Relating test-taking attitudes and skills and stereotype threat effects to the racial gap in cognitive ability test performance. Human Performance. 2003:16; 261-293.

Smith CE, Hopkins RH. Mitigating the impact of stereotypes on academic performance: The effects of cultural identity and attributions for success among African American college students. The Western Journal of Black Studies. 2004:28; 312-321.

**The following 3 manuscripts were excluded, as they did not prime stereotype threat:**

Bonnot V, Croizet J-C. Stereotype internalization and women’s math performance: the role of interference in working memory. Journal of Experimental Social Psychology. 2007:43; 857-866.

Osborne JW. Testing stereotype threat: Does anxiety explain race and sex differences in achievement? Contemporary Educational Psychology. 2001:26; 291-310.

Owens J, Massey DS. Stereotype threat and college academic performance: A latent variables approach. Social Sciences Research. 2011:40; 150-166.

**The following 2 articles were excluded as they did not measure performance:**

Gaines SO, Bagha S, Barrie M, Bhattacharjee T, Boateng Y, Briggs J, et al. Impact of experiences with racism on African-Descent persons’ susceptibility to stereotype threat within the United Kingdom. Journal of Black Psychology. 2011:27; 135-152.

Von Hippel C, Issa M, Ma R, Stokes A. Stereotype threat: Antecedents and consequences for working women. European Journal of Social Psychology. 2011:41; 151-161.

**The following 4 review papers were excluded:**

Beilock, SL, McConnell AR. Stereotype threat and sport: Can athletic performance be threatened? Journal of Sport & Exercise Psychology. 2004:26; 597-609.

Derks, B., Inzlicht, M., & Kang, S. (2008). The neuroscience of stigma and stereotype threat. Group Processes & Intergroup Relations, 11, 163-181.

Shapiro JR, Neuberg SL. From stereotype threat to stereotype threats: Implications of a multi-threat framework for causes, moderators, mediators, consequences, and interventions. Pers Soc Psychol Rev. 2007:11; 107-130.

Smith JL. Understanding the process of stereotype threat: A review of mediational variables and new performance goal directions. Educational Psychology Review. 2004:16; 177-206.

**The following 3 manuscripts were excluded as they examined moderators of stereotype threat:**

Forbes CE, Leitner JB. Stereotype threat engenders neural attentional bias toward negative feedback to undermine performance. Biological Psychology. 2014:102; 98-107.

Kiefer AK, Sekaquaptewa D. Implicit stereotypes and women’s math performance: How implicit gender-math stereotypes influence women’s susceptibility to stereotype threat. Journal of Experimental Social Psycholog. 2007:43; 825-832.

Kiefer AK, Sekaquaptewa D. Implicit stereotypes, gender identification, and math-related outcomes: A prospective study of female college students. Psychological Science. 2007:18; 13-18.

**The following article was excluded as performance was operationalised in a different way to the rationale of the current review:**

Tellhed U, Björklund F. Stereotype threat in salary negotiations is mediated by reservation salary. Personality and Social Psychology. 2011:52; 185-195.
