# Supplementary material for: Twenty Years of Stereotype Threat Research: A Review of Psychological Mediators
Source: PLoS One. 2016 Jan 11;11(1):e0146487. doi: 10.1371/journal.pone.0146487 (PMC4713435; doi:10.1371/journal.pone.0146487)
Supplement: S2 Table — (DOCX) [file pone.0146487.s003.docx]

S2. Table

Summary of affective/subjective, cognitive and motivational mechanisms that have been found to mediate stereotype threat effects.

| Proposed Mediator | Mediation | Population; Task | Authors |
| --- | --- | --- | --- |
| **Affective/Subjective Mechanisms** | | | |
| Non-verbal Anxiety | Complete | Men; childcare, interpersonal skills | Bosson et al. [29] |
| Anxiety; specific self-efficacy | Complete, sequential | Job Applicants; promotion exam | Chung et al. [73] |
| Anxiety; mind-wandering | Complete, sequential | Women; math test | Mrazek et al. [74] |
| Somatic Anxiety | Partial | Students; motor performance | Laurin [75] |
| Anxiety; self-concept | Moderated-mediation | Women; math test | Gerstenberg et al. [76] |
| Individuation Tendencies | Partial | Women; spatial ability test | Keller & Sekaquaptewa [78] |
| Performance expectations | Partial | Women; math test  African-American solders; verbal ability test | Cadinu et al. [80] |
| Performance expectations | Partial | Women; math test | Rosenthal et al. [81] |
| **Cognitive Mechanisms** | | | |
| Working memory | Complete | Women; math test | Schmader & Johns [89] |
| Working memory | Complete | Women; math test | Rydell et al. [92] |
| Emotion Regulation | Complete | Women; math test | Johns et al. [90] |
| Updating | Complete | Women; math test | Rydell et al. [93] |
| Mental load | Complete | Students; Cognitive test | Croizet et al. [94] |
| Thought suppression | Partial | Students; math test | Logel et al. [95] |
| Negative thinking | Complete | Women; math test | Cadinu et al. [96] |
| Cognitive Appraisal | Complete | Students; visuospatial ability test | Berjot et al. [100] |
| Implicit stereotype endorsement | Complete | Children; math test | Galdi et al. [103] |
| **Motivational Mechanisms** | | | |
| Effort | Complete | Older adults; memory recall test | Hess et al. [18] |
| Effort | Complete | Provisional drivers; hazard perception test | Skorich et al. [27] |
| Effort | Complete | Women; visuospatial ability test | Jamieson & Harkins [105] |
| Self-handicapping | Partial | Students; golf-putting | Stone [15] |
| Self-handicapping | Complete | Students; maths test | Keller [109] |
| Dejection | Partial | Women; visuospatial ability test | Keller & Dauenheimer [44] |
| Vigilance | Partial | Students; analytic reasoning | Seibt & Förster [108] |
| Avoidance goals | Complete | Women; math test | Brodish & Devine [112] |
